# Lectures on Diseases of the Skin

**Published:** 1886-12

**Authors:** Henry Wile

**Affiliations:** Lecturer on Dermatology, Atlanta, Ga.


THE ATLANTA MEDICAL AND SURGICAL JOURNAL.

VOL. III. DECEMBER, 1886. NO. 10.

### Original Communications.

### LECTURES ON DISEASES OF THE SKIN.

DELIVERED AT THE ATLANTA MEDICAL COLLEGE DURING THE SESSION OF 1885-6, BY HENRY WILE, M. D., LECTURER ON DERMATOLOGY, ATLANTA, GA.

### LECTURE X.

CLASS II. INFLAMMATIONS—ERYTHEMA MULTIFORME—ERYTHEMA NODOSUM—URTICARIA.

Reported Stenographically for THE ATLANTA MEDICAL AND SURGICAL JOURNAL by W. Kay Tewksbury.

The class of diseases called inflammations is remarkable for the number and variety of diseases it embraces. Though having a common pathology, these diseases present distinct clinical and morphological characteristics according to which they are further classified. The process of inflammation, as considered in a preceding lecture, causes alterations of structure and in general effects the cutaneous tissues in proportion to its cause, location and intensity. It may be confined to certain layers or some particular structure of the skin. Then again, it may be circumscribed or diffuse, involving the entire organ. The process may at one time culminate in the formation of a macule, at another time a papule, vesicle or pustule, or a combination of these lesions. According to the morphological character of the lesions, the diseases are subdivided into macular or erythematous, papular, vesicular and pustular groups. They may be acute or chronic and always give rise in the course of their development to subjective symptoms, as burning and itching, which may be highly distressing to the patient.

ERYTHEMA MULTIFORME is an acute disease occurring in the form of variously sized macules or diffuse erythematous areas which may develop into papules, vesicles or tubercles. The lesions appear suddenly, for the most part, on the back of the hands and feet, about the wrist and ankles, forearms and legs, exceptionally on the face and trunk, and present in the course of their development striking color changes. When in the form of macular lesions the color is at first bright red; in a few days the centre changes to a dark red and finally to a bluish hue. As they increase in size, the periphery is of a bright red, forming a marked contrast with the darker centre. At times the color of the centre gradually becomes normal, and the lesions, developing peripherally, present the appearance of bright red rings, giving rise to *erythema annulare.* Sometimes the macular lesions unite, forming large erythematous patches. As these gradually fade away the border appears irregular or scolloped, it being then called *erythema marginatum.* Several annular lesions developing side by side may finally unite and prsent the form of part circles. This variety is known as *erythema gyratum.* The macules may develop into papules, *erythema papulatum,* several of which coalescing may produce *erythema tuberculosum.*

The papules may develop into vesicles, and especially in the form of concentric rings, or vesicles may develop directly in a spot, then others appearing outside of these and coalescing form a ring. The vesicles in the cetnre dry up and disappearing leave a dark red spot. The lesion continues to progress peripherally until it attains the size varying from a pea to a quarter dollar. The successive appearance and disappearance of vesicles produce a variegated appearance, whence the name *erythema iris*

*et circinatum*. These spots are single or multiple and appear especially around the wrists and forehead, or about the lower extremities. The successive rings of different colors, from dark red to blue and yellow, are produced by the gradual absorption of the pigment from the centre towards the periphery. This variegation advances peripherally as the vesicles disappear; hence we have successive colored rings. This variety of erythema, very marked in its appearance and its course of developement, occurs especially in children and runs its course in from two to six weeks.

The vesicles occasionally develop into blebs, producing *erythema bullosum*. The disease in disappearing leaves pigmented spots, and in case of a vesicular eruption a slight desquamation follows. The development of the lesions is usually attended with slight burning or itching and not infrequently with rheumatic pains in the bones in the vicinity. At times gastric and acute febrile symptoms are also present as complications. The disease runs its course in from one to eight weeks, according to the liability to relapse, which is common. It appears mostly in the spring and fall of the year, and is encountered more in the young of both sexes. It disappears spontaneously.

The exciting causes of erythema multiforme are not clear. According to some it may result from local irritation; according to others it is a reflex process, having been observed to follow gastric and urethral disorders. According to Lewin (charite annalen, 1876, p. 656), the erythema is secondary to an infiltration in the subcutaneous connective tissues. The capillaries are dilated and surrounded in some places with red and white blood corpuseles. The lymph spaces and vessels are also crowded with cells. The diagnosis is established from the peculiar development of the lesions, that is from a central point which, as the process advances peripherally, assumes a dark red then a bluish tint. Also from the location about the hands and feet, the acuteness, brief duration of the process and the multiforme character of the lesions. The prognosis is favorable, as the disease almost invariably ends in spontaneous recovery, leaving slight pigment residue which also slowly fades away.

The treatment, when necessary, is at most soothing and palliative. When vesicles are present or the lesions are irritated, mechanically absorbent powders, as of oxide of zinc, subnitrate of bismuth or starch, may be used.

ERYTHEMA NODOSUM appears in the form of nut to egg-sized nodes or tumor-like bodies, buried in the cutaneous tissues or projecting slightly above the level of the surface and appreciated by palpitation on account of their firm resistant character. Their location is marked at first by a red surface which, as the development of the process goes on, changes to a blue, yellow or a greenish hue. The development is usually accompanied by febrile and gastric symptoms, also severe rheumatic pains in the long bones and neighboring joints. If the disease is in the lower extremities this pain may be so severe as to prevent the patient from moving about. The lesions are painful on pressure and occur for the most part on the lower extremities below the knees, also on the forearm, but rarely on the trunk. They appear in successive crops in varying numbers, scattered about and continue from one to several weeks, disappearing gradually by absorption without suppuration and leaving a light yellowish pigmented spot that continues for a time. The disease runs an acute course and ends spontaneously. The cause is obscure. It effects especially young individuals, even infants, those of weakly constitutions and mostly females, and occurs as a rule in the spring and fall. The pathology of this disease is not clear. The process is regarded as a result of a disturbance of the vaso-motor nerves. Lewin found a dilitation of the capillaries of the papillary body as well as those of the upper layers of the corium, these vessels being surrounded by groups of red and white blood corpuscles, especially the latter. These corpuscles continued around the vessels, extending in the form of stripes to the subcutaneous connective tissue, where they were found in abundance, separating the meshes of the connective tissues. The process is regarded by Lewin and others as an hemorrhagic exudation.

In the diagnosis of this disease the lesions, when few in number, may be mistaken for contusions, but the successive appearance in crops, the red or violaceous color, the localization, together with the accompanying rheumatoid pain, are characteristic.

The prognosis is benign, although the process may be severe in debilitated patients, especially children. Relapses may occur.

The treatment is directed towards the correction of any constitutional disorder, as fever or gastric symptoms. Quinine, some mild laxative and a regulation of the diet is usually sufficient. When the rheumatoid pains are severe salicylic acid in ten to forty grain doses often gives relief. Locally, cold applications of water or ice prove agreeable.

URTICARIA (hives—nettlerash) consists in the sudden development of wheals. The eruption may be local or universal and is attended with an itching, prickling or tingling sensation. The lesions vary in size, being usually the size of a pea; at times they are as large as a silver quarter or silver dollar (giant urticaria). They are of a pinkish or light red color, the larger ones having a pale or yellowish centre and pink periphery. In form they are round, but the larger patches forming by coalition of smaller lesions are irregular and may assume striking shapes, as circles, crescents. Occasionally the skin is so sensitive that by scratching the surface once or twice with the finger nail a line of wheals will make its appearance.

Rarely the lesions are in the form of pin-head to pea-sized papules (urticaria papulosa), occurring especially in children and giving rise to severe itching. There may also be exudations of serum producing vesicles or blebs (urticaria bullosa).

The disease generally runs an acute course, the lesions appearing and disappearing in crops quickly, in the space of minutes or hours, the attack lasting a few days or becoming chronic and lasting for months or years, with or without remission. The acute form may be accompanied by febrile or gastric disturbances.

The causes are manifold, being sometimes clear and standing in direct relation to the disease; at other times they are obscure. They may be divided into internal and external, both producing the same results by irritation of the vaso-motor nerves.

Among the internal causes may be mentioned certain medicinal agents, whose internal administration is occasionally promptly followed by an eruption of urticaria, thus quinia, balsam copaiba, powdered cubebs, morphia, chloral, turpentine, salicylate of sodium, and santonin. Also the ingestion of certain articles of food, as the different shellfish, berries, pickles, sausage, pork, honey, cheese, wines, and the presence of worms in the alimentary tract. Gastric or intestinal symptoms may or may not be present. Cases are on record where an urticarial eruption appeared during an attack of colic, due to the passage of gall-stones. These causes act indirectly and the cutaneons lesions are the result of reflex irritation. In children is this especially the case, where these symptoms are common and usually attributable to improper diet or the method of artificial feeding. The disease is also met with during the period of dentition. It may accompany Bright's disease of the kidneys, diabetes, diseases of the liver and genito-urinary tract; also rheumatism.

Among the external causes may be cited the irritation from the stings of insects, as the mosquito, bedbug, flea, louse and caterpillar. Scratching the lesions serves to develop them and they become at once more prominent.

The lesion of urticaria forms almost a connecting link between a simple hyperæmia and an inflammation. It shows an exudation in the superficial layers of the corium, which, according to Newmann, causes a compression of the blood-vessels and gives rise to a circumscribed œdema and swelling of the cells of the rete mucosum.

The diagnosis of urticaria should present no difficulties, as the lesion, *wheal*, together with the marked subjective symptoms, burning, tingling or prickling, are eminently characteristic. In determining the variety and cause of the eruption, great discrimination must sometimes be exercised, especially when it is associated with disease of internal organs.

In the prognosis one should be guided by the diagnosis as to the form, whether acute or chronic. The former usually subsides as soon as the cause is removed, while the latter may prove persistent and annoying. Relapses are common, but the disease is in nowise serious.

The first object in the treatment should be to discover and remove the cause, external or internal. When the disease is due to the ingestion of certain articles of food or medicines, a purgative is indicated, such as castor oil, olive oil, seidlitz powder, rochelle salts, or a solution of the citrate of magnesia. This may be repeated, if necessary, so as to remove from the alimentary canal all sources of reflex irritation. The diet must be regulated with special reference to this matter. Where other organic causes exist, they should be investigated and remedied. According to one authority salicylate of sodium, in twenty grain doses, is said to abort an attack of urticaria. In the chronic form the sulphate of atropia, $\frac{1}{100}$ grain three times daily, sometimes acts most happily.

The local treatment is directed towards the subjection of the often distressing symptoms of itching, prickling and burning. For this purpose cooling lotions are best adapted. Cold sponge baths often prove effective. Also the following lotions: ether one-half ounce, glycerine two drachms, alcohol four ounces, acetic acid one ounce, rose water seven ounces, carbolic acid one to four drachms to water one pint. Bicarbonate of soda or alum baths, one-half pound to the bath, have an agreeable and soothing effect. When other measures fail, an ointment of tar or sulphur, one-half to one drachm to the ounce of oxide of zinc ointment, will at times be efficient.

